# The Effective Synthesis of *N*-(Arylalkyl)-1-R-4-hydroxy-2,2-dioxo- 1*H*-2λ^6^,1-benzothiazine-3-carboxamides as Promising Analgesics of a New Chemical Class

**DOI:** 10.3797/scipharm.1506-04

**Published:** 2015-07-17

**Authors:** Igor V. Ukrainets, Lidiya A. Petrushova, Sergiy P. Dzyubenko, Galina Sim, Lina A. Grinevich

**Affiliations:** 1 Department of Pharmaceutical Chemistry, National University of Pharmacy, 53 Pushkinska St., 61002, Kharkiv, Ukraine; 2 Department of Pharmaceutical Chemistry, N. I. Pirogov Vinnitsa National Medical University, 56 Pirogov St., Vinnitsa, 21018, Ukraine; 3 Department of Pharmaceutical Chemistry, Far Eastern State Medical University, 35 Murav’eva-Amurskogo St., 680000, Khabarovsk, Russia

**Keywords:** Amidation, Analgesia, Arylalkylamines, Synthesis, Pain syndrome, 4-Hydroxy-2,2-dioxo-1,2-dihydro-2λ^6^,1-benzothiazine-3-carboxamides, 2,1-Benzothiazines

## Abstract

A new, effective preparative method has been proposed and the synthesis of a series of *N*-(arylalkyl)-1-R-4-hydroxy-2,2-dioxo-1*H*-2λ^6^,1-benzothiazine-3-car-boxamides has been carried out. It has been shown that amidation of alkyl 1-R-4-hydroxy-2,2-dioxo-1*H*-2λ^6^,1-benzothiazine-3-carboxylates with arylalkyl-amines in boiling xylene proceeds with good yield and purity to the corresponding *N*-(arylalkyl)-amides. However, the presence of water in the reaction mixture has been shown to cause the formation of specific impurities: *N*-(arylalkyl)-1-R-2,2-dioxo-1*H*-2λ^6^,1-benzothiazin-4-amines. According to the results of the pharmacological studies, powerful analgesics have been found among the substances synthesized.

## Introduction

The idea of creating the “ideal analgesic” was born in the middle of the last century and has not yet found practical implementation, but nowadays it actively continues to attract the attention of scientists of different specialties [1–5]. The undying interest in the given problem becomes quite clear and understandable, just by remembering that the feeling of pain to some extent is familiar to all of us. Appearing as unpleasant, depressing, if not unbearable sensations, pain first signals danger and thus plays an important role in protecting human health and even life. However, this does not mean that it should be borne. Strong and long-lasting pain stimulation exhausts the internal resources of the organism causing serious disorders of its vital functions. Therefore, the fight against pain and pain syndromes is recognized as one of the priority problems of medicine [6].

Modern science offers a wide range of methods for relieving pain and for pain control. However, the main role still belongs to medicines [7], and their improvement is conducted in different ways. For example, the methodology of creating combined drugs has had good results. It has appeared that the simultaneous intake of several substances with different mechanisms of pain suppression provides more profound analgesic effects in general than each component separately [8–10]. An important event of modern pharmacotherapy is the introduction of suitable dosage forms of analgesics – retard tablets and transdermal therapeutic systems. Their single use can maintain the desired concentration of the drug in blood for a long time, and thus an adequate level of analgesia, thereby greatly improving the quality of life of patients with chronic pain [11–13]. The numerous variants of their chemical modification are widely and fruitfully used to optimize certain properties of drugs that are already known [14].

Unfortunately, completely new or innovative analgesics do not often appear. It is not an easy task to find unexplored and, at the same time, promising classes of chemical compounds. Sometimes nature itself gives very useful tips for finding such compounds in substances that are produced by plants or animals and are highly active. These are often familiar to humans with their unique properties, and they are isolated in a pure form and subject to thorough pharmacological testing [15–19]. It is clear that not every study of this kind ends with the creation of a specific drug, however, the importance of the information obtained is undoubted for further research.

**Fig. 1:**
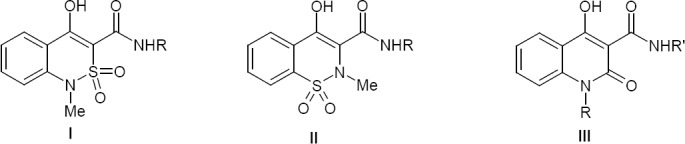
General formula of highly active analgesics [7, 21, 25–29]

Our choice for study subjects is based on a principle that is controversial, but nevertheless frequently used by medicinal chemists: similar substances should exhibit similar biological activity [20]. In other words, we have chosen 4-hydroxy-1-methyl-2,2-dioxo-1*H*-2λ^6^,1-benzothiazine-3-carboxamides **I** (Fig. 1) based on the fact that they are isomers of nonsteroidal anti-inflammatory drugs from the group of oxicams **II**, they differ only with a reciprocal arrangement of atoms of nitrogen and sulfur in the thiazine fragment, and have perfectly proven themselves in medical practice as pain killers [7]. At the same time, amides **I** can be considered as sulfo analogues of 4-hydroxy-2-oxo-1,2-dihydroquinoline-3-carboxamides **III**; powerful analgesics have also been found among them [21]. Finally, considering the fact that due to the lack of efficient methods for synthesis so far, derivatives of 4-hydroxy-2,2-dioxo-1*H*-2λ^6^,1-benzothiazine-3-carboxylic acids remain the class of chemical compounds that is almost unstudied.

**Fig. 2:**
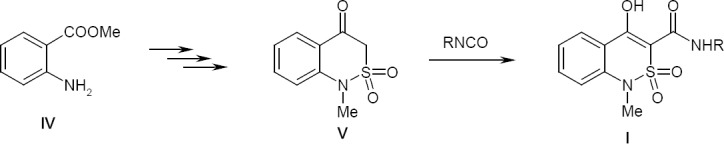
Known method for the synthesis of 4-hydroxy-1-methyl-2,2-dioxo-1*H*-2λ^6^,1-benzothiazine-3-carboxamides **I**.

It should be noted that some of 1-*N*-methyl substituted carboxamides **I** have already been obtained by the reaction of 1-methyl-3,4-dihydro-2,2-dioxo-1*H*-2λ^6^,1-benzothiazin-4-one (**V**) and isocyanates. This four-step synthetic pathway (Fig. 2) was suggested more than 40 years ago [22]. But it is rarely used and usually only if it is necessary to obtain amides **I** as model compounds [23]. Its major disadvantage is low yield at the first two stages of the synthesis of 2,l-benzothiazine **V**. Also, the use of isocyanates, which are often expensive or even unavailable reagents, appears to be the weakest link.

## Results and Discussion

### Chemistry

A completely different three-step scheme of assembling target amides **1a–s** has been proposed, its key stage is amidation of alkyl 1-R-4-hydroxy-2,2-dioxo-1*H*-2λ^6^,1-benzothiazine-3-carboxylates [24] **2a,b** with the primary amines in dry, boiling xylene (Scheme 1). The method has not been worked out yet; however, it initially has the advantage, which is important for any work devoted to the search of the “structure – property” regularities, of the ability to use an unlimited and readily available range of the most diverse amines. This gives very good prospects.

**Sch. 1:**
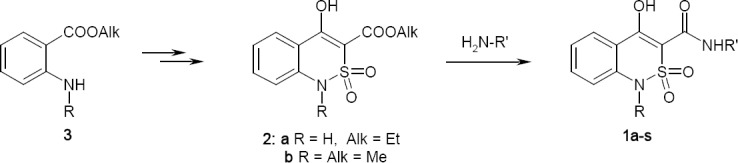
Synthesis of 4-hydroxy-2,2-dioxo-1*H*-2λ^6^,1-benzothiazine-3-carboxamides **1**

In the synthesis of the nearest analogues of drugs of the oxicam series, *N*-hetaryl-4-hydroxy-1-methyl-2,2-dioxo-1*H*-2λ^6^,1-benzothiazine-3-carboxamides [25–27], our method has shown good results. There also are no problems in obtaining anilides [28, 29]. However, the reaction with 1*H*-1,2,4-triazol-5-amine takes place not so unambiguously, and in addition to the corresponding amides it unexpectedly leads to the formation of new condensed heterocyclic systems, triazolopyrimidobenzothiazines [30].

Our research is intended to explain how esters **2a,b** will behave in the reaction with arylalkylamines and most importantly, how it will affect the biological properties of 4-hydroxy-2,2-dioxo-1*H*-2λ^6^,1-benzothiazine-3-carboxamides. The interest of including *N*-(arylalkyl)-substituted derivatives **1** as the objects of study is based primarily on the fact that among 4-hydroxy-2-oxo-1,2-dihydroquinoline-3-carboxamides **III**, similar by their structures, arylalkylamides appeared to be the most powerful pain killers [21, 31–33].

Our attempts to carry out amidation in methanol or ethanol using methyl 4-hydroxy-1-methyl-2,2-dioxo-1*H*-2λ^6^,1-benzothiazine-3-carboxylate (**2b**) and benzylamine were unsuccessful: even after refluxing for 30 h, benzylamide **1e** was not found in the reaction mixture. Unlike highly reactive alkyl 4-hydroxy-2-oxo-1,2-dihydroquinoline-3-carboxylate, which forms the corresponding arylalkylamides in low-boiling alcohols very easily [31–33], their sulfo analogues **2** appeared to be completely inert relative to the arylalkylamines in the same conditions. However, in more severe conditions, (xylene, refluxing), *N*-benzyl-4-hydroxy-1-methyl-2,2-dioxo-1*H*-2λ^6^,1-benzothiazine-3-carbox-amide (**1e**) was synthesized with a good yield (Scheme 2).

**Sch. 2:**
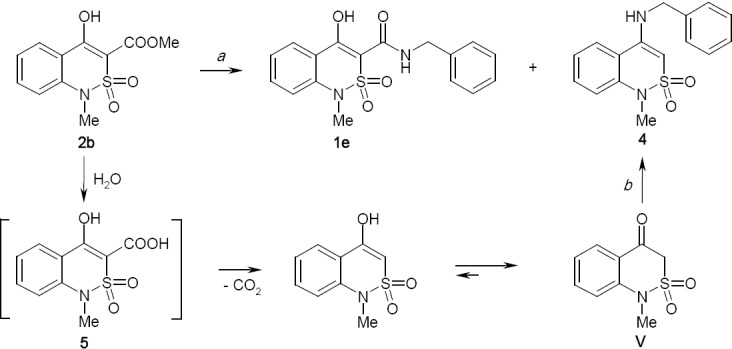
Reaction of ester **2b** and benzylamine. Reagents and conditions: (a) benzylamine, xylene, 150°C, 1 h, 88% (**1e**), 12% (**4**); (b) benzylamine, MeOH, 65°C, 3 h, 96% (4).

The quality control of crude benzylamide **1e** by HPLC has demonstrated that it contains approximately 12% of the impurity, which according to the chromatographic characteristics was not the starting ester **2b**. The ^1^H-NMR spectrum of the above-mentioned sample showed that the benzylamine fragment was present in both amide **1e** and in the impurity detected. Interested in this fact, it was decided to determine the real structure of the minor product of the studied reaction; from this, one can first understand and then eliminate the causes of its formation.

**Fig. 3:**
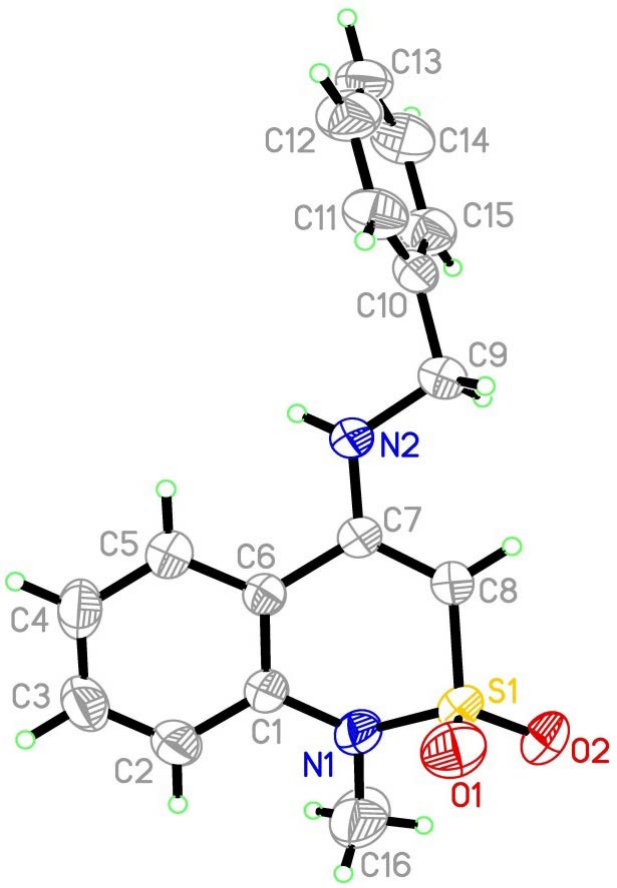
The molecular structure of 4-*N*-benzylsubstituted benzothiazine 4 with atoms represented by thermal vibration ellipsoids of 50% probability.

While slowly evaporating the mother liquor remaining after treatment of a crude benzylamide **1e** with a strongly cooled acetone, we succeeded in growing some single crystals suitable for X-ray diffraction analysis. As a result, it has been clearly identified that the byproduct was *N*-benzyl-1-methyl-2,2-dioxo-1*H*-2λ^6^,1-benzothiazin-4-amine (**4**). Of the features of the spatial structure of this compound (Fig. 3), it may just be noted that its dihydrothiazine heterocycle is in “twist-boat” conformation (folding parameters [34]: S 0.66, Θ 53.4°, Ψ 28.9°). Deviations of atoms S_(1)_ and C_(8)_ from the mean square plane of the remaining atoms of the cycle are −0.95 and −0.37 Å, respectively. Both nitrogen atoms have a pyramidal configuration with a low degree of pyramidicity: N_(1)_- and N_(2)_-centered sums of bond angles are 355° and 356°, respectively. The benzyl substituent is in *sp*-conformation in relation to the endocyclic bond C_(7)_–C_(8)_, and its aromatic ring is in *ap*-conformation in relation to the C_(7)_–N_(2)_ bond and is significantly turned in relation to the N_(2)_–C_(9)_ bond.

In our opinion, the most likely cause of the impurity of 4-benzylamino substituted benzothiazine **4** is the trivial presence of water in one of the reagents or solvent. Earlier, we mentioned several times the peculiarity of alkyl 4-hydroxy-2-oxo-1,2-dihydroquinoline-3-carboxylate to react more readily with water than with amines at 120–150°C. At this temperature, hydrolysis has serious competition for amidation that all water present in the reaction mixture is involved in the formation of the corresponding quinoline-3-carboxylic acids. Those, in turn, quickly undergo decarboxylation. As a result, target 4-hydroxy-2-oxo-1,2-dihydroquinoline-3-carboxamides are contaminated by specific impurities, 4-hydroxy-quinolin-2-ones [35, 36].

It is obvious that there is the same side reaction in amidation of benzothiazine esters **2**; moreover, they are hydrolyzed much easier than their 2-carbonyl analogues, and we even have not had a chance to isolate the intermediate benzothiazine-3-carboxylic acids **5** yet, due to their extreme instability [37]. The only difference is that benzothiazine analogues of 4-hydroxyquinolin-2-ones unsubstituted in position 3 exist predominantly in 4-ketoform **V**, and it is more significant in our case, in contrast to them and their synthetic precursors, esters **2**, they possess a high reactivity in relation to *N*-nucleophiles. Therefore, byproducts of the reactions of benzothiazine esters **2** and arylalkylamines are not 4-hydroxy-(oxo)- benzothiazines **V**, but their 4-amino derivatives **4**. It is this mechanism of appearance of the given impurities in *N*-(arylalkyl)-1-R-4-hydroxy-2,2-dioxo-1*H*-2λ^6^,1-benzothiazine-3-carboxamides **1a-s** that is confirmed by formation of 4-*N*-benzylsubstituted benzothiazine **4** in the reaction of methyl-3,4-dihydro-2,2-dioxo-1*H*-2λ^6^,1-benzothiazin-4-one (**V**) and benzylamine in boiling methanol, i.e. under the conditions that are definitely unsuitable for amidation of esters **2**. Differences in the rates of competing main and side reactions are still not very great. Otherwise, even a small amount of water quickly depleted on the hydrolysis of ester **2** would return again in the reaction mixture after the formation of 4-amino derivative **4**, restarting the process of hydrolysis, etc. Thus, the amidation would be largely or even completely suppressed, but it does not correspond to the experimental data.

After determining the cause of specific impurities’ appearance, it was not difficult to minimize an undesirable side reaction. For this purpose it is sufficient to remove water not only from the solvent, but from both reagents as well. Esters **2a,b** are nonhygroscopic, they were dried in the air at room temperature. Commercial xylene and arylalkylamines were dried with anhydrous CaCl_2_ in granules and КОН in tablets, respectively, followed by distillation. According to HPLC data in a new sample of benzylamide **1e** obtained from reagents previously prepared, the content of the impurity of 4-amino derivative **4** decreased up to 0.05%, and it could be neglected. As a result, according to this method the target *N*-(arylalkyl)-1-R-4-hydroxy-2,2-dioxo-1*H*-2λ^6^,1-benzothiazine-3-carboxamides **1a-s** were obtained with good yields and purity. Naturally, cyclohexylmethyl-substituted derivative **1s** does not belong to arylalkylamides, but it is of interest as a hydrogenated analogue of benzylamide **1e**. All the products were characterized by elemental analysis, ^1^H- and ^13^C-NMR data. It should be noted as a distinctive feature of the ^1^H-NMR spectra of amides **1a-s** that due to rapid proton exchange in the amide group, *N*-methylene protons of benzyl- and phenethyl-amide fragments appear exclusively as singlets or triplets, respectively, in all cases instead of the classic doublets or quartets. For the same reason, the amide protons of these fragments give broad singlets.

### Evaluation of the Analgesic Activity

Analysis of the experimental data presented in Table 1 shows that our choice of *N*-(arylalkyl)-1-R-4-hydroxy-2,2-dioxo-1*H*-2λ^6^,1-benzothiazine-3-carboxamides as targets of research is fully justified; each and all compounds exhibited analgesic properties. Moreover, it is possible to assert unequivocally that derivatives **1a-d** unsubstituted in position 1 are not of practical interest because their activity is too weak. However, the 1-*N-*methyl substituent cardinally changes the situation. Benzylamide **1e**, for example, exceeds Meloxicam more than twice in the similar dose by the potency of its analgesic effect. Introduction of substituents in the aromatic nucleus of the benzyl fragment regardless of their position and nature is accompanied by a decrease in activity, moreover, in a fairly wide range. So, if in the case of 4-chloro- (**1h**) and 4-methylbenzylamide **1k** it can be classified as minor, whereas 2-methyl- (**1i**) and 4-methoxy (**1m**) groups inactivate the basic molecule almost completely. The removal of the phenyl nucleus from the amide nitrogen atom for another methylene unit causes a similar effect as evidenced by the extremely weak analgesic effect of phenethylamide **1o**. Interestingly, in this group of compounds, the substituents in the phenyl nucleus is markedly enhanced the activity, i.e. they cause quite the opposite effect compared to benzylamides. The elongation of the hydrocarbon chain separating the aromatic nucleus and the amide nitrogen atom by up to three methylene groups (3-phenylpropylamide **1r**), as well as hydrogenation of the benzyl substituent (cyclohexylmethylamide **1s**), lead to a decrease in activity approximately twice although it is still quite high at the level of Meloxicam.

**Tab. 1:**
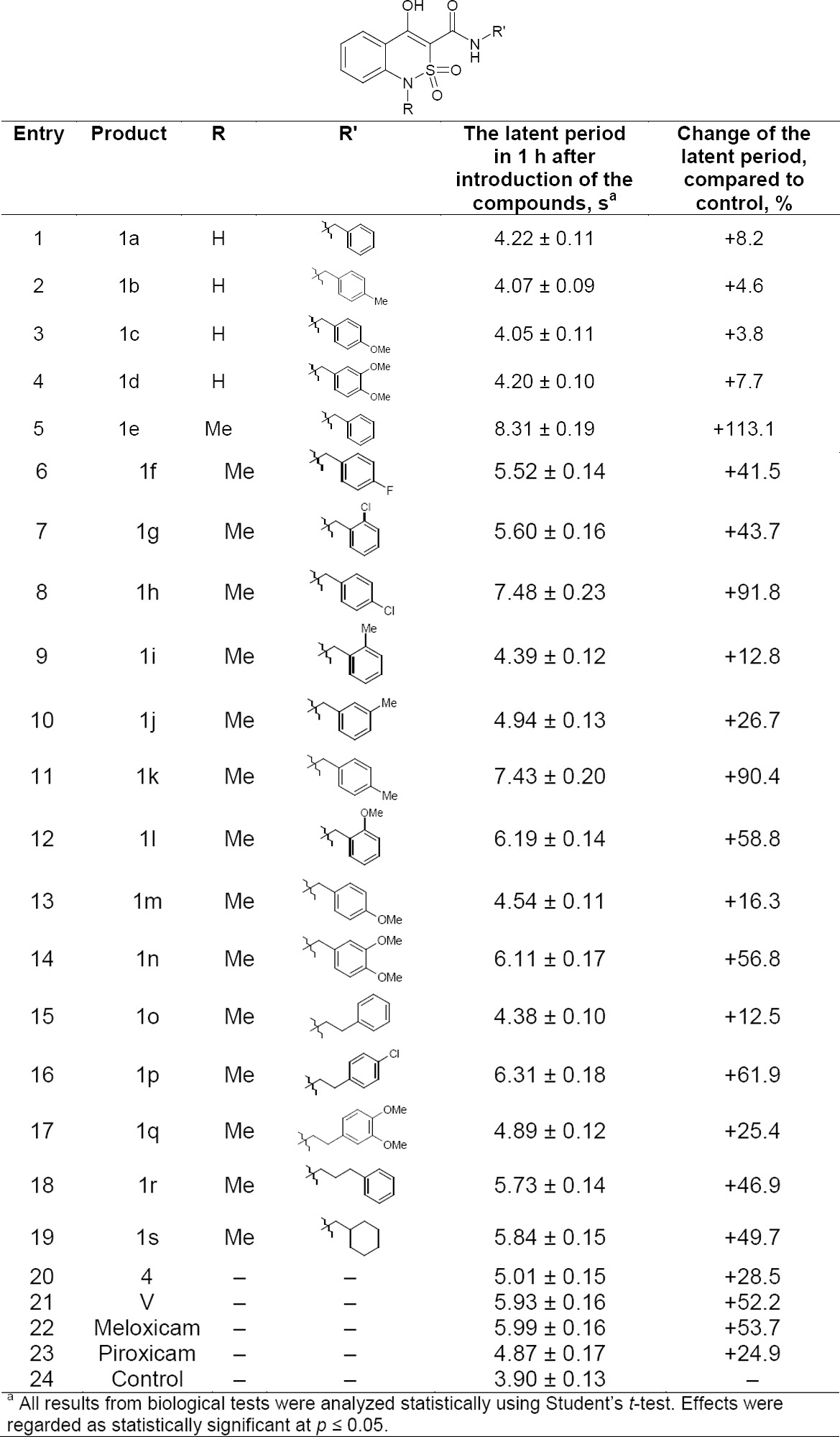
The analgesic activity of arylalkylamides **1a–s**, benzothiazine **4, V**, and reference drugs.

It is interesting to note a pronounced analgesic effect of the byproduct of the studied reaction, *N*-benzyl-1-methyl-2,2-dioxo-1*H*-2λ^6^,1-benzothiazin-4-amine (**4**) and especially its synthetic precursor, l-methyl-3,4-dihydro-2,2-dioxo-1*H*-2λ^6^,1-benzothiazin-4-one (**V**).

## Experimental

### Chemistry

^1^H- and ^13^C-NMR spectra were acquired on a Varian Mercury-400 instrument (400 and 100 MHz, respectively) in DMSO-d_6_ with TMS as internal standard. The chemical shift values were recorded on a *δ* scale and the coupling constants (*J*) in hertz. The following abbreviations were used in reporting spectra: s = singlet, d = doublet, t = triplet, quin = quintet, m = multiplet. Elemental analysis was performed on a Euro Vector EA-3000 Microanalyzer. Melting points were determined in a capillary using a Stuart SMP10 digital melting point apparatus. The reaction mixtures obtained after the reaction of ester **2b** with benzylamine were analyzed on a modular Bischoff HPLC system with Lambda 1010 spectrophotometric detector (Bischoff Analysentechnik GmbH). The chromatographic conditions were: ProntoSIL 120-5-CN column of 4.0 × 250 mm; the sorbent particle size was 5 μm; the mobile phase flow rate was 1 ml/min; the column temperature was 40°C; the injection volume was 5 μl; detection at 254 nm; the mobile phase composition was MeCN–H_2_O (87.3:12.7%). The synthesis of alkyl 1-R-4-hydroxy-2,2-dioxo-1*H*-2λ^6^,1-benzothiazine-3-carboxylates (**2**) was carried out by the method in the study [24].

### General Procedure for the Synthesis of N-(arylalkyl)-1-R-4-hydroxy-2,2-dioxo-1H-2λ^6^,1-benzothiazine-3-carboxamides (1a–s)

A mixture of ester **2** (0.01 mol), arylalkylamine (0.01 mol), and dry xylene (2 ml) was kept for 1 h at 150°C in a liquid metal bath using a suitable air-cooled distilling column that allowed us to distill off the methanol or ethanol formed without removing the xylene solvent. The reaction mixture was cooled, EtOH (5 ml) was added, and the mixture was left for several hours at room temperature. The crystalline amide **1** that precipitated was filtered off, washed with cold EtOH, dried, and recrystallized from EtOH. Arylalkylamides **1a**–**s** were colorless crystals.

#### N-Benzyl-4-hydroxy-2,2-dioxo-1H-2λ^6^,1-benzothiazine-3-carboxamide (1a)

Yield: 94%; mp 183-185°C; ^1^H-NMR (400 MHz, DMSO-d_6_): δ 16.28 (s, 1H, 4-OH), 12.15 (br. s, 1H, SO_2_NH), 8.38 (br. s, 1H, CONH), 7.93 (d, 1H, *J* = 8.0 Hz, H-5), 7.62 (t, 1H, *J* = 7.7 Hz, H-7), 7.30 (t, 1H, *J* = 7.6 Hz, H-6), 7.26-7.15 (m, 6H, H-8 + Ph), 4.48 (s, 2H, NCH_2_). Anal. Calcd. for C_16_H_14_N_2_O_4_S: C, 58.17; H, 4.27; N, 8.48; S 9.71%. Found: C, 58.24; H, 4.33; N, 8.56; S 9.66%.

#### 4-Hydroxy-N-(4-methylbenzyl)-2,2-dioxo-1H-2λ^6^,1-benzothiazine-3-carboxamide (1b)

Yield: 91%; mp 186-188°C; ^1^H-NMR (400 MHz, DMSO-d_6_): δ 16.22 (br. s, 1H, 4-OH), 12.12 (br. s, 1H, SO_2_NH), 8.36 (br. s, 1H, CONH), 7.91 (d, 1H, *J* = 8.0 Hz, H-5), 7.63 (t, 1H, *J* = 7.7 Hz, H-7), 7.31-7.08 (m, 6H, H-6,8,2’,3’,5’,6’), 4.49 (s, 2H, NCH_2_), 2.25 (s, 3H, Me). Anal. Calcd. for C_17_H_16_N_2_O_4_S: C, 59.29; H, 4.68; N, 8.13; S 9.31%. Found: C, 59.35; H, 4.77; N, 8.18; S 9.33%.

#### 4-Hydroxy-N-(4-methoxybenzyl)-2,2-dioxo-1H-2λ^6^,1-benzothiazine-3-carboxamide (1c)

Yield: 93%; mp 180-182°C; ^1^H-NMR (400 MHz, DMSO-d_6_): δ 16.29 (br. s, 1H, 4-OH), 12.18 (br. s, 1H, SO_2_NH), 8.34 (br. s, 1H, CONH), 7.91 (d, 1H, *J* = 8.0 Hz, H-5), 7.62 (t, 1H, *J* = 7.8 Hz, H-7), 7.32-7.218 (m, 3H, H-6,2’,6’), 7.14 (d, 1H, *J* = 8.3 Hz, H-8), 6.89 (d, 2H, *J* = 8.0 Hz, H-3’,5’), 4.45 (s, 2H, NCH_2_), 3.70 (s, 3H, OMe); Anal. Calcd. for C_17_H_16_N_2_O_5_S: C, 56.66; H, 4.48; N, 7.77; S 8.90%. Found: C, 56.70; H, 4.55; N, 7.71; S 8.82%.

#### N-(3,4-Dimethoxybenzyl)-4-hydroxy-2,2-dioxo-1H-2λ^6^,1-benzothiazine-3-carboxamide (1d)

Yield: 90%; mp 189-191°C; ^1^H-NMR (400 MHz, DMSO-d_6_): δ 16.21 (br. s, 1H, 4-OH), 12.16 (br. s, 1H, SO_2_NH), 8.31 (br. s, 1H, CONH), 7.91 (d, 1H, *J* = 8.0 Hz, H-5), 7.63 (t, 1H, *J* = 7.7 Hz, H-7), 7.26 (t, 1H, J = 7.5 Hz, H-6), 7.14 (d, 1H, *J* = 8.3 Hz, H-8), 6.97 (s, 1H, H-2’), 6.91 (d, 1H, *J* = 8.3 Hz, H-5’), 6.84 (d, 1H, *J* = 8.3 Hz, H-6’), 4.46 (s, 2H, NCH_2_), 3.72 (s, 3H, OMe), 3.70 (s, 3H, OMe). Anal. Calcd. for C_18_H_18_N_2_O_6_S: C, 55.38; H, 4.65; N, 7.18; S 8.21%. Found: C, 55.32; H, 4.72; N, 7.24; S 8.29%.

#### N-Benzyl-4-hydroxy-1-methyl-2,2-dioxo-1H-2λ^6^,1-benzothiazine-3-carboxamide (1e)

Yield: 92%; mp 143-145-°C; ^1^H-NMR (400 MHz, DMSO-d_6_): δ 16.39 (s, 1H, 4-OH), 8.59 (br. s, 1H, NH), 8.04 (d, 1H, *J* = 7.9 Hz, H-5), 7.79 (t, 1H, *J* = 7.8 Hz, H-7), 7.52 (d, 1H, *J* = 8.3 Hz, H-8), 7.41 (t, 1H, *J* = 7.7 Hz, H-6), 7.35-7.28 (m, 5H, Ph), 4.60 (s, 2H, NCH_2_), 3.45 (s, 3H, NMe). ^13^C-NMR (100 MHz, DMSO-d_6_): δ 169.8 (C-OH), 166.4 (C=O), 140.8, 138.8, 135.4, 129.2, 128.1, 127.9, 127.1, 124.5, 119.1, 118.9, 103.5 (C-3), 43.5 (NCH_2_), 32.2 (NCH_3_). Anal. Calcd. for C_17_H_16_N_2_O_4_S: C, 59.29; H, 4.68; N, 8.13; S 9.31%. Found: C, 59.35; H, 4.76; N, 8.04; S 9.23%.

#### N-(4-Fluorobenzyl)-4-hydroxy-1-methyl-2,2-dioxo-1H-2λ^6^,1-benzothiazine-3-carboxamide (1f)

Yield: 89%; mp 161-163°C; ^1^H-NMR (400 MHz, DMSO-d_6_): δ 16.42 (s, 1H, 4-OH), 8.60 (br. s, 1H, NH), 8.01 (d, 1H, *J* = 8.0 Hz, H-5), 7.77 (t, 1H, *J* = 7.7 Hz, H-7), 7.49 (d, 1H, *J* = 8.3 Hz, H-8), 7.44-7.36 (m, 3H, H-6,2’,6’), 7.18 (t, 2H, *J* = 8.6 Hz, H-3’,5’), 4.64 (s, 2H, NCH_2_), 3.42 (s, 3H, NMe). ^13^C-NMR (100 MHz, DMSO-d_6_): δ 169.8 (C-OH), 166.4 (C=O), 163.2/160.8 (d, ^1^*J*_C-F_ = 246 Hz, C-4’), 140.8, 135.5, 135.1, 136.3/130.2 (d, ^3^*J*_C-F_ = 8.2 Hz, C-2’,6’), 127.1, 124.5, 119.0, 118.9, 116.0/115.8 (d, ^2^*J*_C-F_ = 21.5 Hz, C-3’,5’), 103.5 (C-3), 42.9 (NCH_2_), 32.2 (NCH_3_). Anal. Calcd. for C_17_H_15_FN_2_O_4_S: C, 56.35; H, 4.17; N, 7.73; S 8.85%. Found: C, 56.38; H, 4.24; N, 7.80; S 8.78%.

#### N-(2-Chlorobenzyl)-4-hydroxy-1-methyl-2,2-dioxo-1H-2λ^6^,1-benzothiazine-3-carboxamide (1g)

Yield: 91%; mp 167-169°C; ^1^H-NMR (400 MHz, DMSO-d_6_): δ 16.10 (s, 1H, 4-OH), 8.41 (br. s, 1H, NH), 8.04 (d, 1H, *J* = 8.0 Hz, H-5), 7.74 (t, 1H, *J* = 7.7 Hz, H-7), 7.46-7.28 (m, 6H, H-6,8,2’,3’,5’,6’), 4.67 (s, 2H, NCH_2_), 3.47 (s, 3H, NMe). ^13^C-NMR (100 MHz, DMSO-d_6_): δ 169.7 (C-OH), 166.6 (C=O), 140.8, 135.8, 135.5, 132.7, 130.0, 129.7, 129.3, 128.1, 127.1, 124.5, 119.0, 118.9, 103.5 (C-3), 41.6 (NCH_2_), 32.2 (NCH_3_). Anal. Calcd. for C_17_H_15_ClN_2_O_4_S: C, 53.90; H, 3.99; N, 7.39; S 8.46%. Found: C, 53.86; H, 4.06; N, 7.32; S 8.39%.

#### N-(4-Chlorobenzyl)-4-hydroxy-1-methyl-2,2-dioxo-1H-2λ^6^,1-benzothiazine-3-carboxamide (1h)

Yield: 95%; mp 138-140°C; ^1^H-NMR (400 MHz, DMSO-d_6_): δ 16.32 (s, 1H, 4-OH), 8.63 (br. s, 1H, NH), 8.01 (d, 1H, *J* = 8.0 Hz, H-5), 7.77 (t, 1H, *J* = 7.8 Hz, H-7), 7.49 (d, 1H, *J* = 8.4 Hz, H-8), 7.43-7.39 (m, 3H, H-6,2’,6’), 7.35 (d, 2H, *J* = 8.2 Hz, H-3’,5’), 4.55 (s, 2H, NCH_2_), 3.42 (s, 3H, NMe). ^13^C-NMR (100 MHz, DMSO-d_6_): δ 169.8 (C-OH), 166.5 (C=O), 140.8, 138.0, 135.5, 132.4, 130.0, 129.1, 127.1, 124.5, 119.0, 118.9, 103.5 (C-3), 42.9 (NCH_2_), 32.2 (NCH_3_). Anal. Calcd. for C_17_H_15_ClN_2_O_4_S: C, 53.90; H, 3.99; N, 7.39; S 8.46%. Found: C, 53.98; H, 4.06; N, 7.47; S 8.37%.

#### 4-Hydroxy-1-methyl-N-(2-methylbenzyl)-2,2-dioxo-1H-2λ^6^,1-benzothiazine-3-carboxamide (1i)

Yield: 85%; mp 170-172°C; ^1^H-NMR (400 MHz, DMSO-d_6_): δ 16.28 (s, 1H, 4-OH), 8.37 (br. s, 1H, NH), 8.02 (d, 1H, *J* = 8.0 Hz, H-5), 7.79 (t, 1H, *J* = 7.6 Hz, H-7), 7.50 (d, 1H, *J* = 8.4 Hz, H-8), 7.39 (t, 1H, *J* = 7.6 Hz, H-6), 7.27-7.15 (m, 4H, H-3’,4’,5’,6’), 4.57 (s, 2H, NCH_2_), 3.43 (s, 3H, NMe). ^13^C-NMR (100 MHz, DMSO-d_6_): δ 169.7 (C-OH), 166.4 (C=O), 140.8, 136.4, 136.2, 135.5, 130.8, 127.9, 127.8, 127.1, 126.6, 124.5, 119.0, 118.9, 103.4 (C-3), 41.6 (NCH_2_), 32.2 (NCH_3_), 19.3 (2’-CH_3_). Anal. Calcd. for C_18_H_18_N_2_O_4_S: C, 60.32; H, 5.06; N, 7.82; S 8.95%. Found: C, 60.37; H, 5.11; N, 7.87; S 9.03%.

#### 4-Hydroxy-1-methyl-N-(3-methylbenzyl)-2,2-dioxo-1H-2λ^6^,1-benzothiazine-3-carboxamide (1j)

Yield: 88%; mp 126-128°C; ^1^H-NMR (400 MHz, DMSO-d_6_): δ 16.40 (s, 1H, 4-OH), 8.49 (br. s, 1H, NH), 8.02 (d, 1H, *J* = 7.9 Hz, H-5), 7.76 (t, 1H, *J* = 7.6 Hz, H-7), 7.48 (d, 1H, *J* = 8.2 Hz, H-8), 7.38 (t, 1H, *J* = 7.5 Hz, H-6), 7.24 (t, 1H, *J* = 7.2 Hz, H-5’), 7.18 (s, 1H, H-2’), 7.13 (d, 1H, *J* = 7.2 Hz, H-6’), 7.08 (d, 1H, *J* = 7.1 Hz, H-4’), 4.54 (s, 2H, NCH_2_), 3.42 (s, 3H, NMe), 2.29 (s, 3H, Me). ^13^C-NMR (100 MHz, DMSO-d_6_): δ 169.8 (C-OH), 166.4 (C=O), 140.8, 138.7, 138.3, 135.4, 129.1, 128.8, 128.5, 127.1, 125.2, 124.4, 119.0, 118.8, 103.4 (C-3), 43.5 (NCH_2_), 32.1 (NCH_3_), 21.7 (3’-CH_3_). Anal. Calcd. for C_18_H_18_N_2_O_4_S: C, 60.32; H, 5.06; N, 7.82; S 8.95%. Found: C, 60.38; H, 5.13; N, 7.85; S 9.01%.

#### 4-Hydroxy-1-methyl-N-(4-methylbenzyl)-2,2-dioxo-1H-2λ^6^,1-benzothiazine-3-carboxamide (1k)

Yield: 87%; mp 131-133°C; ^1^H-NMR (400 MHz, DMSO-d_6_): δ 16.42 (s, 1H, 4-OH), 8.51 (br. s, 1H, NH), 8.02 (d, 1H, *J* = 8.0 Hz, H-5), 7.78 (t, 1H, *J* = 7.6 Hz, H-7), 7.50 (d, 1H, *J* = 8.3 Hz, H-8), 7.40 (t, 1H, *J* = 7.5 Hz, H-6), 7.26 (d, 2H, *J* = 7.0 Hz, H-2’,6’), 7.17 (d, 2H, *J* = 7.0 Hz, H-3’,5’), 4.53 (s, 2H, NCH_2_), 3.43 (s, 3H, NMe), 2.29 (s, 3H, Me). ^13^C-NMR (100 MHz, DMSO-d_6_): δ 169.8 (C-OH), 166.4 (C=O), 140.8, 137.0, 135.7, 135.4, 129.7, 128.2, 127.1, 124.5, 119.1, 118.9, 103.4 (C-3), 43.3 (NCH_2_), 32.2 (NCH_3_), 21.4 (3’-CH_3_). Anal. Calcd. for C_18_H_18_N_2_O_4_S: C, 60.32; H, 5.06; N, 7.82; S 8.95%. Found: C, 60.24; H, 4.95; N, 7.73; S 8.87%.

#### 4-Hydroxy-1-methyl-N-(2-methoxybenzyl)-2,2-dioxo-1H-2λ^6^,1-benzothiazine-3-carboxamide (1l)

Yield: 90%; mp 165-167°C; ^1^H-NMR (400 MHz, DMSO-d_6_): δ 16.32 (s, 1H, 4-OH), 8.42 (br. s, 1H, NH), 8.01 (d, 1H, *J* = 8.0 Hz, H-5), 7.77 (t, 1H, *J* = 7.6 Hz, H-7), 7.50 (d, 1H, *J* = 8.3 Hz, H-8), 7.39 (t, 1H, *J* = 7.4 Hz, H-6), 7.30 (t, 1H, *J* = 7.5 Hz, H-4’), 7.23 (d, 1H, *J* = 7.3 Hz, H-6’), 7.04 (d, 1H, *J* = 8.0 Hz, H-3’), 6.94 (t, 1H, *J* = 7.2 Hz, H-5’), 4.55 (s, 2H, NCH_2_), 3.85 (s, 3H, OMe), 3.43 (s, 3H, NMe). ^13^C-NMR (100 MHz, DMSO-d_6_): δ 169.7 (C-OH), 166.2 (C=O), 157.6 (C-2’), 140.8, 135.5, 129.6, 128.8, 127.1, 125.7, 124.5, 121.1, 119.0, 118.9, 111.5, 103.3 (C-3), 56.1 (OCH_3_), 39.0 (NCH_2_), 32.2 (NCH_3_). Anal. Calcd. for C_18_H_18_N_2_O_5_S: C, 57.74; H, 4.85; N, 7.48; S 8.56%. Found: C, 57.67; H, 4.80; N, 7.42; S 8.64%.

#### 4-Hydroxy-1-methyl-N-(4-methoxybenzyl)-2,2-dioxo-1H-2λ^6^,1-benzothiazine-3-carboxamide (1m)

Yield: 87%; mp 119-121°C; ^1^H-NMR (400 MHz, DMSO-d_6_): δ 16.43 (s, 1H, 4-OH), 8.46 (br. s, 1H, NH), 8.01 (d, 1H, *J* = 7.9 Hz, H-5), 7.76 (t, 1H, *J* = 7.6 Hz, H-7), 7.49 (d, 1H, *J* = 8.2 Hz, H-8), 7.38 (t, 1H, *J* = 7.5 Hz, H-6), 7.30 (d, 2H, *J* = 8.2 Hz, H-2’,6’), 6.92 (d, 2H, *J* = 8.2 Hz, H-3’,5’), 4.49 (s, 2H, NCH_2_), 3.73 (s, 3H, OMe), 3.41 (s, 3H, NMe). ^13^C-NMR (100 MHz, DMSO-d_6_): δ 169.8 (C-OH), 166.3 (C=O), 159.2 (C-4’), 140.9, 135.4, 130.7, 129.7, 127.1, 124.5, 119.1, 118.9, 114.6, 103.4 (C-3), 55.7 (OCH_3_), 43.0 (NCH_2_), 32.2 (NCH_3_). Anal. Calcd. for C_18_H_18_N_2_O_5_S: C, 57.74; H, 4.85; N, 7.48; S 8.56%. Found: C, 57.69; H, 4.78; N, 7.41; S 8.62%.

#### N-(3,4-Dimethoxybenzyl)-4-hydroxy-1-methyl-2,2-dioxo-1H-2λ^6^,1-benzothiazine-3-carboxamide (1n)

Yield: 91%; mp 160-162°C; ^1^H-NMR (400 MHz, DMSO-d_6_): δ 16.41 (s, 1H, 4-OH), 8.46 (br. s, 1H, NH), 8.04 (d, 1H, *J* = 8.0 Hz, H-5), 7.79 (t, 1H, *J* = 7.6 Hz, H-7), 7.51 (d, 1H, *J* = 8.4 Hz, H-8), 7.40 (t, 1H, *J* = 7.6 Hz, H-6), 7.03 (s, 1H, H-2’), 6.96 (d, 1H, *J* = 8.0 Hz, H-5’), 6.90 (d, 1H, *J* = 8.0 Hz, H-6’), 4.52 (s, 2H, NCH_2_), 3.77 (s, 3H, OMe), 3.75 (s, 3H, OMe), 3.44 (s, 3H, NMe). ^13^C-NMR (100 MHz, DMSO-d_6_): δ 169.7 (C-OH), 166.3 (C=O), 149.3, 148.8, 140.8, 135.4, 131.0, 127.1, 124.5, 120.5, 119.0, 118.9, 112.5, 112.4, 103.4 (C-3), 56.2 (OCH_3_), 56.1 (OCH_3_), 43.3 (NCH_2_), 32.2 (NCH_3_). Anal. Calcd. for C_19_H_20_N_2_O_6_S: C, 56.43; H, 4.98; N, 6.93; S 7.93%. Found: C, 56.48; H, 5.06; N, 7.00; S 7.85%.

#### 4-Hydroxy-1-methyl-N-phenethyl-2,2-dioxo-1H-2λ^6^,1-benzothiazine-3-carboxamide (1o)

Yield: 86%; mp 107-109°C; ^1^H-NMR (400 MHz, DMSO-d_6_): δ 16.48 (s, 1H, 4-OH), 8.07 (br. s, 1H, NH), 8.01 (d, 1H, *J* = 8.0 Hz, H-5), 7.77 (t, 1H, *J* = 7.7 Hz, H-7), 7.49 (d, 1H, *J* = 8.4 Hz, H-8), 7.39 (t, 1H, *J* = 7.5 Hz, H-6), 7.31 (t, 2H, *J* = 7.4 Hz, H-2’,6’), 7.26 (d, 2H, *J* = 7.4 Hz, H-3’,5’), 7.21 (t, 1H, *J* = 7.2 Hz, H-4’), 3.60 (t, 2H, *J* = 6.8 Hz, NCH_2_), 3.41 (s, 3H, NMe), 2.87 (t, 2H, *J* = 7.2 Hz, NCH_2_CH_2_). ^13^C-NMR (100 MHz, DMSO-d_6_): δ 169.8 (C-OH), 166.4 (C=O), 140.8, 139.4, 135.4, 129.4, 129.1, 127.1, 127.0, 124.5, 119.1, 119.0, 103.2 (C-3), 41.6 (NCH_2_), 35.4 (NCH_2_CH_2_), 32.3 (NCH_3_). Anal. Calcd. for C_18_H_18_N_2_O_4_S: C, 60.32; H, 5.06; N, 7.82; S 8.95%. Found: C, 60.33; H, 5.01; N, 7.93; S 8.88%.

#### N-(4-Chlorophenethyl)-4-hydroxy-1-methyl-2,2-dioxo-1H-2λ^6^,1-benzothiazine-3-carboxamide (1p)

Yield: 90%; mp 122-124°C; ^1^H-NMR (400 MHz, DMSO-d_6_): δ 16.44 (s, 1H, 4-OH), 8.07 (br. s, 1H, NH), 8.00 (d, 1H, *J* = 7.9 Hz, H-5), 7.76 (t, 1H, *J* = 7.8 Hz, H-7), 7.49 (d, 1H, *J* = 8.4 Hz, H-8), 7.39 (t, 1H, *J* = 7.5 Hz, H-6), 7.34 (d, 2H, *J* = 8.1 Hz, H-3’,5’), 7.28 (d, 2H, *J* = 8.1 Hz, H-2’,6’), 3.59 (t, 2H, *J* = 6.8 Hz, NCH_2_), 3.40 (s, 3H, NMe), 2.87 (t, 2H, *J* = 7.2 Hz, NCH_2_CH_2_). ^13^C-NMR (100 MHz, DMSO-d_6_): δ 169.8 (C-OH), 166.4 (C=O), 140.8, 138.4, 135.4, 131.7, 131.3, 129.0, 127.1, 124.5, 119.0, 118.9, 103.2 (C-3), 41.3 (NCH_2_), 34.6 (NCH_2_CH_2_), 32.3 (NCH_3_). Anal. Calcd. for C_18_H_17_ClN_2_O_4_S: C, 55.03; H, 4.36; N, 7.13; S 8.16%. Found: C, 54.96; H, 4.29; N, 7.17; S 8.11%.

#### N-(3,4-Dimethoxyphenethyl)-4-hydroxy-1-methyl-2,2-dioxo-1H-2λ^6^,1-benzothiazine-3-carboxamide (1q)

Yield: 89%; mp 115-117°C; ^1^H-NMR (400 MHz, DMSO-d_6_): δ 16.51 (s, 1H, 4-OH), 8.06 (br. s, 1H, NH), 8.00 (d, 1H, *J* = 7.9 Hz, H-5), 7.77 (t, 1H, *J* = 7.6 Hz, H-7), 7.49 (d, 1H, *J* = 8.3 Hz, H-8), 7.39 (t, 1H, *J* = 7.4 Hz, H-6), 6.89 (d, 1H, *J* = 8.3 Hz, H-5’), 6.84 (s, 1H, H-2’), 6.76 (d, 1H, *J* = 8.3 Hz, H-6’), 3.75 (s, 3H, OMe), 3.71 (s, 3H, OMe), 3.58 (t, 2H, *J* = 6.7 Hz, NCH_2_), 3.40 (s, 3H, NMe), 2.80 (t, 2H, *J* = 6.9 Hz, NCH_2_CH_2_). ^13^C-NMR (100 MHz, DMSO-d_6_): δ 169.8 (C-OH), 166.4 (C=O), 149.3, 148.0, 140.8, 135.4, 131.8, 127.1, 124.5, 121.2, 119.0, 118.9, 113.2, 112.6, 103.2 (C-3), 56.2 (OCH_3_), 55.9 (OCH_3_), 41.8 (NCH_2_), 34.9 (NCH_2_CH_2_), 32.2 (NCH_3_). Anal. Calcd. for C_20_H_22_N_2_O_6_S: C, 57.40; H, 5.30; N, 6.69; S 7.66%. Found: C, 57.34; H, 5.25; N, 6.61; S 7.72%.

#### 4-Hydroxy-1-methyl-N-(3-phenylpropyl)-2,2-dioxo-1H-2λ^6^,1-benzothiazine-3-carboxamide (1r)

Yield: 82%; mp 89-91°C; ^1^H-NMR (400 MHz, DMSO-d_6_): δ 16.53 (s, 1H, 4-OH), 8.09 (br. s, 1H, NH), 8.02 (d, 1H, *J* = 7.9 Hz, H-5), 7.78 (t, 1H, *J* = 7.7 Hz, H-7), 7.50 (d, 1H, *J* = 8.3 Hz, H-8), 7.40 (t, 1H, *J* = 7.5 Hz, H-6), 7.30 (t, 2H, *J* = 7.4 Hz, H-2’,6’), 7.22 (d, 2H, *J* = 7.4 Hz, H-3’,5’), 7.17 (t, 1H, *J* = 7.3 Hz, H-4’), 3.43 (s, 3H, NMe), 3.38 (t, 2H, *J* = 6.4 Hz, NCH_2_), 2.63 (t, 2H, *J* = 7.3 Hz, NCH_2_CH_2_CH_2_), 1.88 (quin. 2H, *J* = 7.1 Hz, NCH_2_CH_2_CH_2_). ^13^C-NMR (100 MHz, DMSO-d_6_): δ 169.7 (C-OH), 166.4 (C=O), 141.9, 140.8, 135.4, 129.0, 128.9, 127.1, 126.5, 124.4, 119.1, 118.9, 103.3 (C-3), 39.8 (NCH_2_), 33.1 (CH_2_-Ph), 32.2 (NCH_3_), 30.9 (NCH_2_CH_2_). Anal. Calcd. for C_19_H_20_N_2_O_4_S: C, 61.27; H, 5.41; N, 7.52; S 8.61%. Found: C, 61.35; H, 5.47; N, 7.47; S 8.55%.

#### N-Cyclohexylmethyl-4-hydroxy-1-methyl-2,2-dioxo-1H-2λ^6^,1-benzothiazine-3-carboxamide (1s)

Yield: 80%; mp 93-95°C; ^1^H-NMR (400 MHz, DMSO-d_6_): δ 16.57 (s, 1H, 4-OH), 8.04 (d, 1H, *J* = 8.0 Hz, H-5), 7.98 (br. s, 1H, NH), 7.79 (t, 1H, *J* = 7.8 Hz, H-7), 7.52 (d, 1H, *J* = 8.4 Hz, H-8), 7.41 (t, 1H, *J* = 7.4 Hz, H-6), 3.44 (s, 3H, NMe), 3.26 (d, 2H, *J* = 6.2 Hz, NCH_2_), 1.74-1.60 (m, 6H, 3,4,5-CH_2_), 1.27-0.91 (m, 5H, 2-CH_2_CHCH_2_-6). ^13^C-NMR (100 MHz, DMSO-d_6_): δ 169.7 (C-OH), 166.4 (C=O), 140.7, 135.4, 127.1, 124.5, 118.9, 118.8, 103.3 (C-3), 45.8 (NCH_2_), 37.8 (CH), 32.2 (NCH_3_), 30.8 (2,6-CH_2_), 26.6 (4-CH_2_), 25.9 (3,5-CH_2_). Anal. Calcd. for C_17_H_22_N_2_O_4_S: C, 58.27; H, 6.33; N, 7.99; S 9.15%. Found: C, 58.36; H, 6.39; N, 8.04; S 9.11%.

### N-Benzyl-1-methyl-2,2-dioxo-1H-2λ^6^,1-benzothiazin-4-amine (4-(Benzylamino)-1-methyl-2λ^6^,1-benzothiazine-2,2(1H)-dione, 4)

Benzylamine (1.18 g, 0.011 mol) was added to a solution of 1-methyl-3,4-dihydro-2,2-dioxo-1*H*-2λ^6^,1-benzothiazin-4-one (**V**) (2.11 g, 0.01 mol) in methanol (10 ml) and heated at reflux for 3 h. The reaction mixture was cooled, diluted by adding cold water, and brought to pH ~ 4 by adding acetic acid and was used in the isolation of 4-*N*-benzylsubstituted benzothiazine **4**. The precipitate formed was filtered off, washed with cold water, and dried. Yield: 2.88 g (96%), colorless crystals of mp 167-169°C (acetone). ^1^H-NMR (400 MHz, DMSO-d_6_): δ 7.90 (d, 1H, *J* = 8.0 Hz, H-5), 7.61-7.47 (m, 2H, H-7 + NH), 7.39-7.20 (m, 7H, H-6,8 + Ph), 5.63 (s, 1H, H-3), 4.33 (d, 2H, *J* = 5.9 Hz, NCH_2_), 3.19 (s, 3H, NMe). Anal. Calcd. for C_16_H_16_N_2_O_2_S: C, 63.98; H, 5.37; N, 9.33; S 10.67%. Found: C, 64.04; H, 5.44; N, 9.28; S 10.63%.

### X-ray Structural Analysis

Crystal data for *N*-benzyl-1-methyl-2,2-dioxo-1*H*-2λ^6^,1-benzothiazin-4-amine (**4**): C_16_H_16_N_2_O_2_S, colorless, monoclinic (acetone). At 20°C *a* = 9.558(1), *b* = 7.5316(6), *c* = 20.639(2) Å, *β* = 91.865(9)°, *V* = 1484.9(3) Å^3^, *M*_r_ = 300.37, *Z* = 4, space group P2_1_/n, *d*_calc_ = 1.478 g/cm^3^, µ (MoK*_α_*) = 0.261 mm^−1^, F(000) = 624. The unit cell parameters and intensities of 12499 reflections (4326 independent, *R*_int_ = 0.061) were measured on an Xcalibur-3 Diffractometer (MoK*_α_* radiation, CCD detector, graphite monochromator, ω-scanning to 2θ_max_ = 60°). The structure was solved by the direct method using the *SHELXTL* program package [38]. The hydrogen atom positions were revealed by differential synthesis of electron density and refined according to the “rider” model with *U*_iso_ = *nU*_eq_ for the nonhydrogen atom bonded to a given hydrogen atom (*n* = 1.5 for methyl group, *n* = 1.2 for the rest of the hydrogen atoms). The amino group hydrogen atom participating in hydrogen bonds were refined in isotropic approximation. The structure was refined using *F*^2^ full-matrix least-squares analysis in the anisotropic approximation for non-hydrogen atoms to *wR*_2_ = 0.153 for 4261 reflections (*R*_1_ = 0.064 for 2221 reflections with *F* > 4*σ* (*F*), *S* = 0.932). CCDC 1053043 contains the supplementary crystallographic data for this paper. These data can be obtained free of charge from the Cambridge Crystallographic Data Centre via www.ccdc.cam.ac.uk/data_request/cif.

### Pharmacology

#### Analgesic *Test*

The analgesic activity of the synthesized compounds was studied using the model of the thermal tail-flick procedure in white rats (Tail Immersion Test) by comparing those with similar structure, Piroxicam (Jenapharm, Germany) and Meloxicam (Boehringer Ingelheim, Germany), on [39] enabling judgment of the central effect on the nociceptive system. For this purpose, the rat’s tail tip was immersed in a water bath heated to 54°C, and the latent period of the tail withdrawal (immersion) expressed in seconds was determined. The analgesic effect (in %) was assessed by the change of the latent period in 1 hour after introduction of the test substances and reference drugs. Seven experimental animals were involved to obtain statistically reliable results (the significance level of the confidence interval accepted in this work was *p* ≤ 0.05) in testing each of arylalkylamides **1a–s**, reference drugs, and control. All substances under research, Piroxicam, and Meloxicam were introduced orally in the form of fine aqueous suspensions stabilized with Tween-80 in the dose of 20 mg/kg. The animals of the control group received an equivalent amount of water with Tween-80.

## Conclusion

It has been experimentally proven that the direction of the reaction of alkyl 1-R-4-hydroxy-2,2-dioxo-1*H*-2λ^6^,1-benzothiazine-3-carboxylates and arylalkylamines in boiling xylene can vary significantly: in the presence of water *N*-(arylalkyl)-1-R-2,2-dioxo-1*H*-2λ^6^,1-benzothiazin-4-amines are notably formed, whereas in the anhydrous conditions the corresponding *N*-(arylalkyl)-1-R-4-hydroxy-2,2-dioxo-1*H*-2λ^6^,1-benzothiazine-3-carbox-amides are obtained with good yields. The regularities of the revealed “structure – activity” relationship are discussed. According to the results of the pharmacological study conducted, some compounds that deserve a deeper and more detailed pharmacological study have been found; among them is *N*-benzyl-4-hydroxy-1-methyl-2,2-dioxo-1*H*-2λ^6^,1-benzothiazine-3-carboxamide which is certainly the structure-leader as a new promising pain killer.

## References

[1] Carter HM (1946). The ideal analgesic in labor and childbirth. Med World (New York).

[2] Bentley KW (1964). The relief of pain - the search for the ideal analgesic. Endeavour.

[3] Todd WM (1984). The search for the ideal analgesic. Acta Anaesthesiol Belg.

[4] Tao PL, Law PY, Loh HH (2010). Search for the “ideal analgesic” in pain treatment by engineering the mu-opioid receptor. IUBMB Life.

[5] Mathuram Thiyagarajan U, Bagul A, Nicholson ML (2012). Pain management in laparoscopic donor nephrectomy: a review. Pain Res Treat.

[6] Goldberg D S, McGee S J (2011). Pain as a global public health priority. BMC Public Health.

[7] Kleemann A, Engel J, Kutscher B, Reichert D (2008). Pharmaceutical Substances: Syntheses, Patents, Applications of the most relevant APIs.

[8] Zor F, Ozturk S, Bilgin F, Isik S, Cosar A (2010). Pain relief during dressing changes of major adult burns: ideal analgesic combination with ketamine. Burns.

[9] Smith HS (2008). Combination opioid analgesics. Pain Physician.

[10] Madhusudhan SK (2013). Novel analgesic combination of tramadol, paracetamol, caffeine and taurine in the management of moderate to moderately severe acute low back pain. J Orthop.

[11] Kern KU, Krings D, Waldmann-Rex S (2014). [Tapentadol prolonged release improves analgesia, functional impairment and quality of life in patients with chronic pain who have previously received oxycodone/naloxone]. MMW Fortschr Med.

[12] Szkutnik-Fiedler D, Sawicki W, Balcerkiewicz M, Mazgalski J, Grabowski T, Grześkowiak E (2014). Biopharmaceutical evaluation of new slow release tablets obtained by hot tableting of coated pellets with tramadol hydrochloride. Acta Pol Pharm.

[13] Kesmati M, Torabi M (2014). Interaction between Analgesic Effect of Nano and Conventional size of Zinc Oxide and Opioidergic System Activity in Animal Model of Acute Pain. Basic Clin Neurosci.

[14] Kubinyi H (2006). Looking ups of the New Compounds-leaders for Creation of Drugs. Russian Chem J.

[15] Alonso D, Khalil Z, Satkunanthan N, Livett B G (2003). Drugs from the sea: conotoxins as drug leads for neuropathic pain and other neurological conditions. Mini Rev Med Chem.

[16] Gibbs WW (1996). A new way to spell relief: v-e-n-o-m. A toxin from killer sea snails promises a better painkiller. Sci Am.

[17] Andreev YA, Kozlov SA, Koshelev SG, Ivanova EA, Monastyrnaya MM, Kozlovskaya EP, Grishin EV (2008). Analgesic compound from sea anemone Heteractis crispa is the first polypeptide inhibitor of vanilloid receptor 1 (TRPV1). J Biol Chem.

[18] Zwart R, Strotton M, Ching J, Astles PC, Sher E (2014). Unique pharmacology of heteromeric α7β2 nicotinic acetylcholine receptors expressed in Xenopus laevis oocytes. Eur J Pharmacol.

[19] Dulu TD, Kanui TI, Towett PK, Maloiy GM, Abelson KS (2014). The effects of oxotremorine, epibatidine, atropine, mecamylamine and naloxone in the tail-flick, hot-plate, and formalin tests in the naked mole-rat (Heterocephalus glaber). In Vivo.

[20] Martin YC, Kofron JI, Traphagen LM (2002). Do structurally similar molecules have similar biological activity?. J Med Chem.

[21] Ukrainets IV, Gorokhova OV, Jaradat NA, Petrushova LA, Mospanova EV, Savchenkova LV, Kuz'min VE, Lyahovsky AV, Racz GB, Noe CE (2014). 4-Hydroxyquinolin-2-ones and their close structural analogues as a new source of highly effective pain-killers. Pain and Treatment.

[22] Lombardino JG (1972). Preparation of some 4-hydroxyl-l-methyl-1*H*-2,l-benzothiazine-3-carboxanilide 2,2-dioxides. J Heterocycl Chem.

[23] Ciske FL, Genin MJ, Lee BH, Schnute ME, Vaillancourt VA (2003). Heterocycle carboxamides as antiviral agents. U.S. Patent 6,559,145.

[24] Ukrainets IV, Petrushova LA, Dzyubenko SP (2013). 2,1-Benzothiazine 2,2-dioxides. 1. Synthesis, structure, and analgesic activity of 1-R-4-hydroxy-2,2-dioxo-1*H*-2λ^6^,1-benzothiazine-3-carboxylic acid esters. Chem Heterocycl Compd.

[25] Ukrainets IV, Petrushova LA, Dzyubenko SP, Sim G (2014). 2,1-Benzothiazine 2,2-dioxides. 3. 4-Hydroxy-1-methyl-2,2-dioxo-*N*-(1,3-thiazol-2-yl)-1*H*-2λ^6^,1-benzothiazine-3-carboxamides - a new group of potential analgetics. Chem Heterocycl Compd.

[26] Ukrainets IV, Petrushova LA, Dzyubenko SP, Liu Y (2014). 2,1-Benzothiazine 2,2-dioxides. 4. Synthesis, structure, and analgesic properties of 4-hydroxy-1-methyl-2,2-dioxo-*N*-(pyridin-2-yl)-1*H*-2λ^6^,1-benzothiazine-3-carboxamides. Chem Heterocycl Compd.

[27] Ukrainets IV, Petrushova LA, Dzyubenko SP, Grinevich LA (2014). Synthesis and analgesic activity of N-(benzothiazol-2-yl)-4-hydroxy-2,2-dioxo-1*H*-2λ^6^,1-benzothiazine-3-carboxamides. Zh Org Farm Khim [J Org Pharm Chem].

[28] Ukrainets IV, Petrushova LA, Dzyubenko SP (2014). Methyl-substituted anilides of 4-hydroxy-1-methyl-2,2-dioxo-1*H*-2λ^6^,1-benzothiazine-3-carboxylic acid. Synthesis, spectral characteristics and biological properties. Zh Org Farm Khim [J Org Pharm Chem].

[29] Petrushova LA, Ukrainets IV, Dzyubenko SP, Grinevich LA (2015). Synthesis and the biological activity of 4-hydroxy-2,2-dioxo-1*H*-2λ^6^,1-benzothiazin-3-carboxylic acids trifluoromethyl-substituted anilides. Zh Org Farm Khim [J Org Pharm Chem].

[30] Ukrainets IV, Petrushova LA, Sim G, Bereznyakova NL (2015). 2,1-Benzothiazine 2,2-dioxides. 10. Reaction of alkyl 1-R-4-hydroxy-2,2-dioxo-1*H*-2λ^6^,1-benzothiazine-3-carboxylates with 1*H*-1,2,4-triazol-5-amine. Chem Heterocycl Compd.

[31] Ukrainets IV, Gorokhova OV, Andreeva KV (2013). Transformation of 3-(3-arylalkylcarbamoyl-4-hydroxy-2-oxo-1,2-dihydroquinolin-1-yl)propanenitriles into amides and acids. Russian J Org Chem.

[32] Ukrainets IV, Mospanova EV, Jaradat NA, Bevz OV, Turov AV (2012). 4-Hydroxy-2-quinolones. 204. Synthesis, bromination, and analgetic properties of 1-allyl-4-hydroxy-6,7-dimethoxy-2-oxo-1,2-dihydroquinoline-3-carboxylic acid arylalkylamides. Chem Heterocycl Compd.

[33] Ukrainets IV, Gorokhova OV, Andreeva KV, Davidenko AA (2014). *N*-Benzyl-1-(2-cyanoethyl)-4-hydroxy-2-oxo-1,2-dihydro-3-quinolinecarboxamides as promising analgesics. Zh Org Farm Khim [J Org Pharm Chem].

[34] Zefirov NS, Palyulin VA, Dashevskaya EE (1990). Stereochemical studies. XXXIV. Quantitative description of ring puckering via torsional angles. The case of six-membered rings. J Phys Org Chem.

[35] Ukrainets IV, Bevz OV, Mospanova EV, Savchenkova LV, Yankovich SI (2012). 4-Hydroxy-2-quinolones. 202. Synthesis, chemical and biological properties of 4-hydroxy-6,7-dimethoxy-2-oxo-1,2-dihydroquinoline-3-carboxylic acid alkylamides. Chem Heterocycl Compd.

[36] Ukrainets IV, Golik MYu, Shemchuk OL, Kravchenko VM (2013). Synthesis and diuretic properties of *N*-aryl-6-hydroxy-2-methyl-4-oxo-2,4-dihydro-1*H*- pyrrolo[3,2,1-*ij*]quinoline-5-carboxamides with electron-acceptor substituents in the anilide fragment. Zh Org Farm Khim [J Org Pharm Chem].

[37] Ukrainets IV, Petrushova LA, Dzyubenko SP, Liu Y (2014). 2,1-Benzothiazine 2,2-dioxides. 5. Hydrolysis of alkyl 1-R-4-hydroxy-2,2-dioxo-1*H*-2λ^6^,1-benzothiazine-3-carboxylates. Chem Heterocycl Compd.

[38] Sheldrick GM (2008). A short history of *SHELX* Acta Crystallogr, Sect A: Found. Crystallogr.

[39] Vogel H G (2008). Drug Discovery and Evaluation: Pharmacological Assays.

